# Delirium, Frailty, and Mortality: Interactions in a Prospective Study of Hospitalized Older People

**DOI:** 10.1093/gerona/glx214

**Published:** 2017-11-01

**Authors:** Melanie Dani, Lucy H Owen, Thomas A Jackson, Kenneth Rockwood, Elizabeth L Sampson, Daniel Davis

**Affiliations:** 1 Department of Medicine for the Elderly, Hammersmith Hospital, London, UK; 2 University College London Hospitals NHS Foundation Trust, London, UK; 3 Institute of Inflammation and Ageing, University of Birmingham, UK; 4 Department of Medicine, Dalhousie University, Halifax, Nova Scotia, Canada; 5 Marie Curie Palliative Care Research Department, UCL Division of Psychiatry, London, UK; 6 MRC Unit for Lifelong Health and Ageing at UCL, London, UK

**Keywords:** Outcomes, Longevity, Risk factors

## Abstract

**Background:**

It is unknown whether the association between delirium and mortality is consistent for individuals across the whole range of health states. A bimodal relationship has been proposed, where delirium is particularly adverse for those with underlying frailty, but may have a smaller effect (perhaps even protective) if it is an early indicator of acute illness in fitter people. We investigated the impact of delirium on mortality in a cohort simultaneously evaluated for frailty.

**Methods:**

We undertook an exploratory analysis of a cohort of consecutive acute medical admissions aged ≥70. Delirium on admission was ascertained by psychiatrists. A frailty index (FI) was derived according to a standard approach. Deaths were notified from linked national mortality statistics. Cox regression was used to estimate associations between delirium, frailty, and their interactions on mortality.

**Results:**

The sample consisted of 710 individuals. Both delirium and frailty were independently associated with increased mortality rates (delirium: HR 2.4, 95% CI 1.8–3.3, *p* < .01; frailty (per *SD*): HR 3.5, 95% CI 1.2–9.9, *p* = .02). Estimating the effect of delirium in tertiles of FI, mortality was greatest in the lowest tertile: tertile 1 HR 3.4 (95% CI 2.1–5.6); tertile 2 HR 2.7 (95% CI 1.5–4.6); tertile 3 HR 1.9 (95% CI 1.2–3.0).

**Conclusion:**

Although delirium and frailty contribute to mortality, the overall impact of delirium on admission appears to be greater at lower levels of frailty. In contrast to the hypothesis that there is a bimodal distribution for mortality, delirium appears to be particularly adverse when precipitated in fitter individuals.

Delirium, characterized by a fluctuating disturbance in arousal, attention, and cognition secondary to an acute medical condition, is common, affecting 18%–35% of general medical inpatients, 8%–17% of older patients attending emergency departments, and 51% of patients in postacute care (1–3). It is associated with increased length of stay, institutionalization, and progression of dementia (4–7).

Delirium is widely understood to be associated with mortality, with an overall HR = 1.9 consistent across a number of studies identified in a systematic review (4). This meta-analysis included observational studies adjusting for chronic comorbidity or acute illness, though none accounted for both. Therefore, an important unanswered question is whether the association remains independent of acute and chronic health factors that might otherwise drive mortality (8). Indeed, a more nuanced understanding of delirium and mortality is relevant given the proposal that the relationship may be bimodal (9). That is, although delirium may have catastrophic outcomes in some patients, for others, it may be an early indicator of acute illness leading to earlier recognition and treatment, perhaps even being protective.

Accounting for underlying frailty may provide an insight into the relationship between delirium and mortality because frailty itself is so closely related to risk of both mortality and delirium. One view of frailty describes the gradual accumulation of deficits as individuals’ age, which results in loss of physiological reserve (physical, mental, and functional) and increased vulnerability to insults (10). Taking baseline functional status and chronic comorbidity into account can resolve the issue of “unmeasured heterogeneity”—the factors that increase risk despite the same level of acute illness (11).

Our aim was to investigate the effect of delirium and frailty on mortality in a large cohort of acutely unwell adults, setting out to answer the following questions (i): Is delirium associated with mortality, even after adjusting for underlying frailty? (ii) Is there an interaction between delirium and frailty? (iii) Is the relationship with frailty and mortality linear across the range of frailty states and does this change according to the presence of delirium?

## Methods

We undertook an exploratory analysis of a cohort prospectively ascertaining outcomes from acutely hospitalized elders. Participants were recruited as previously described (12). Briefly, all patients aged ≥70 years consecutively admitted to the acute medical unit between June 2007 and December 2007 were screened for inclusion. Exclusion criteria were (i) admission length of under 48 hours and (ii) insufficient English to be assessed for cognitive and mental status. The Royal Free Hospitals NHS Trust Ethics Committee gave ethical approval (06/Q0501/31).

## Outcomes

The study was notified of all deaths through linkage with the UK Office for National Statistics for up to 3 years following the index admission.

## Exposures

### Delirium

All participants were evaluated on admission by trained psychiatrists. Formal cognitive testing included the Mini-Mental State Examination (13) and delirium was defined using the Confusion Assessment Method algorithm (14). Key symptoms such as inattention were identified through the “serial 7s” or “W-O-R-L-D backwards” tasks. Other items such as acute onset, disorganized thinking and altered level of consciousness, and degree of fluctuation of these symptoms were ascertained through clinical assessment which included information from ward staff and the medical chart.

### Frailty

A 31-item frailty index (FI) was constructed according to a standardized procedure (15), where the following variables were included: comorbidity (13 variables), examination findings (5 variables), laboratory findings (9 variables), and functional status (4 variables) (Table 1). These variables were selected to encompass the full range of acute and chronic health factors that could account for any observed association with mortality. All items were given a binary score (0 = no deficit, 1 = deficit present). For each participant, an FI score was calculated by dividing the number of deficits present by the denominator of 31 maximum deficits, resulting in a score between 0 and 1. For example, for an individual with 10 deficits present out of 31, their FI score would be 10/31 = 0.32. Across several iterations of FI in several hundred datasets, the usual upper limit of frailty observed asymptotically approaches 0.70. In our dataset, data were not missing for more than 6% in each variable.

**Table 1.** Frailty Index Variables

	Frequency (%)
Comorbidities	
** **Congestive heart failure	21
** **Dementia	24
** **Diabetes	20
** **COPD	17
** **Metastatic disease	6
** **Peripheral vascular disease	7
** **Severe liver disease ** **Any prior tumor	12 16
** **Peptic ulcer disease	9
** **History of MI	28
** **History of CVA	27
** **Psychiatric history	21
** **Alcohol consumption > 1 unit/wk	27
Examination findings	
** **Heart rate (bpm) <70 or >109	34
** **Respiratory rate (breaths/min) <12 or >24	15
** **Temperature (^o^C) <36 or >38.4	10
** **Glasgow coma score ≤13	13
** **MAP (mmHg) <70 or >109	28
Laboratory findings	
** **Packed cell volume (%) <30 or >45.9	18
** **Potassium (mMol/L) <3.5 or >5.4	13
** **Creatinine (μmol/L) <60 or >140	37
** **Sodium (mmol/L) <130 or >149	13
** **White cells (×10^9^/L) <3 or >14.9	21
** **Platelets (×10^9^/L) <150 or >400	21
** **Urea (mmol/L) <2.5 or >7	62
** **CRP (mg/dL) >5	73
** **Albumin (g/L) <35 or >55	19
Functional status	
** **Care home resident	28
** **Urinary and/or faecal incontinence	23
** **Pressure sores present	9
** **Polypharmacy (>4 medications)	52

### Statistical Analysis

Proportional hazards for mortality were assessed in a series of Kaplan–Meier plots and Cox regression models, where outcome was date of death. Postestimation procedures included Schoenfeld residuals for checking assumptions of proportionality. Multiplicative interactions between delirium and FI score were assessed in order to estimate the association of delirium and mortality with respect to underlying frailty, using α = .1 as a threshold for type 1 errors. This approach has been justified by quantifying the gains in power using a less stringent α for samples of this size where interactions between dichotomous (delirium status) and continuous (FI) variables are being considered (16). Linearity of any association with mortality across the distribution of frailty, restricted cubic splines were fitted (four knots), plotting log-hazard ratio for mortality against FI score, stratified by delirium status. Stata version 14.1 was used for all statistical procedures.

## Results

The sample contained 710 individuals, with a mean age of 83.1 years (standard deviation 7.41), and 59% were female (Supplementary Table 1). A diagnosis of dementia was present in 42%. At the end of the 3-year follow-up, 340 individuals remained alive and 370 had died (median follow-up 5 months, IQR 1 to 17 months). The prevalence of delirium on admission was 10.3% (*n* = 73). The prevalence of deficits on index items ranged from 6% (metastatic disease) to 73% (CRP > 5) (Table 1). The mean FI score was 0.23 (*SD* 0.096, upper limit 0.55), with a broadly normal distribution (Supplementary Figure 1).

### Delirium, Frailty, and Mortality

**Figure 1.**
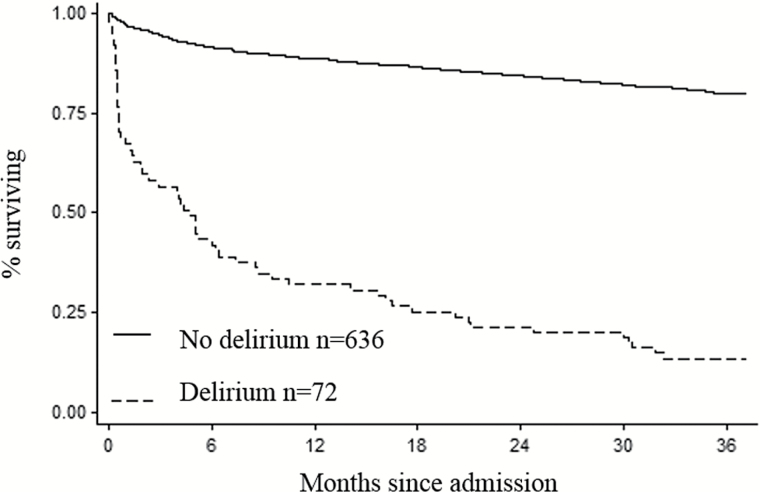
Kaplan–Meier curves showing survival of cohort, by delirium status, adjusted by age and sex.

Delirium was strongly associated with mortality, with 59 (81%) dying within the next 3 years, compared with 311 (49%) without delirium. Tertiles of FI score (first 0–0.19; second 0.20–0.26; third 0.27–0.55) also showed increasing associations with mortality (first 48%; second 51%; third 60%). Both delirium and frailty were crudely associated with increased hazard for death (delirium: 2.4, 95% CI 1.8–3.2; FI [per *SD*]; HR 5.9 [95% CI 2.1–16]). This remained the case with a multivariable model including both terms adjusted by age and sex (delirium: HR 2.4, 95% CI 1.8–3.3, *p* < .01; frailty [per SD]: HR 3.5, 95% CI 1.2–9.9, *p* = .02; Table 2). Kaplan–Meier survival curves (adjusted by age and sex), according to delirium status, demonstrated worse survival for those with delirium (Figure 1).

**Table 2.** Survival Analysis Showing the Associations Between Delirium, Frailty, and Mortality

	Univariable				Multivariable			
	Hazard ratio	95% CI		*p*	Hazard ratio	95% CI		*p*
Delirium	2.44	1.85	3.23	<.01	2.37	1.78	3.15	<.01
Frailty index	5.90	2.14	16.2	<.01	3.37	1.18	9.60	.02

Note: *N* = 708, all models adjusted by age and sex.

Mean FI score = 0.23 (*SD* 0.09).

### Interactions Between Delirium and Frailty

We estimated the effect of delirium on mortality according to each tertile of FI. There was an inverse gradient of association, where stronger associations were observed in the fittest group (tertile 1 HR 3.4 [95% CI 2.1–5.6]; tertile 2 HR 2.7 [95% CI 1.5–4.6]; tertile 3 HR 1.9 [95% CI 1.2–3.0]). The delirium–frailty interaction was statistically significant if α = .1 (*p* = .07).

### Linearity of Mortality Gradients in Relation to Delirium Status

**Figure 2.**
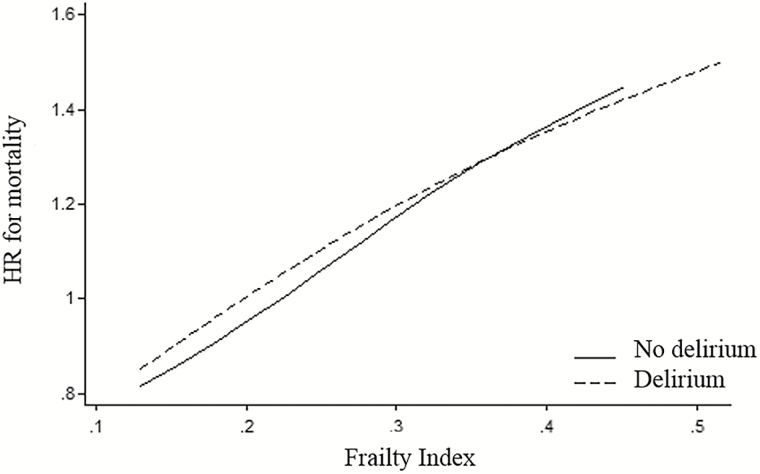
Relationship between frailty and mortality, by delirium status restricted cubic splines modeling relationship between frailty and mortality. The linearity suggests a continuous relationship between frailty and mortality. When stratified by delirium, the lines intersect suggesting greater effects with delirium at lower levels of mortality (*p* = .07).

Restricted cubic splines of the log-HR for mortality against FI score were linear across the four knots, giving no indication that mortality is driven by a particular portion of the frailty continuum (Figure 2). When additionally accounting for delirium status, the splines remained linear, though crossed over to suggest delirium may have greater associations with mortality at lower degrees of frailty and lower associations at higher degrees of frailty (*p* = .07; Figure 2).

## Discussion

We found that on admission, both delirium and frailty were independently associated with increased risk of death, beyond that expected from all the acute and chronic health factors measured in this study. Moreover, delirium and frailty appeared to interact in such a way that although delirium increases the risk of death at all levels of frailty, the relative impact of this association was greatest in fitter patients. The relationship between frailty and mortality was linear, suggesting that mortality is not being driven by a subset of individuals. Delirium status had a small influence on these relationships. Taken together, our results suggest that delirium itself independently confers a mortality risk, and this risk applies whatever the underlying degree of frailty. Our data illustrate some of the advantages of using a FI in studying cognitive disorders by quantifying a multidimensional assessment, thereby allowing other “non-neurological factors” that nevertheless contribute to patient outcomes (eg nutritional status, polypharmacy, electrolyte abnormalities) to be considered (17).

Our findings should be treated with caution. Data were collected from a single site, albeit a large London secondary care hospital with unselected medical admissions from a population of 1.2 million people and high generalizability. Secondly, despite a standardized protocol and training procedure, more than one rater performed the psychiatric evaluation, possibly introducing inter-rater variability. Thirdly, patients were assessed for delirium on admission only (defined as within 72 hours of admission). Any patients developing delirium after this point would not be included in this analysis, possibly underestimating the effects of delirium. Strengths included large sample size and validated assessments undertaken by specialist diagnosticians. Moreover, a wide variety of variables were available to construct a broad FI. The specific inclusion of physiological and laboratory items may generally relate to acute illness, and it is becoming clear that use of such measures is nonetheless informative for use in a FI (18). Although functional items were under-represented, for the requirements of this specific analysis, the combination of acute and chronic health factors made for a particularly robust FI measure.

To understand why the relative risk is higher in fit patients compared with frail patients, the insult causing delirium in fit individuals needs to be large, in order to overcome their physiological and cognitive reserve. Another reason for delirium in fitter individuals may be a distinct neurological precipitant which could drive the poorer prognosis in these patients. Both frailty and delirium are associated with vulnerability and lack of physiological and cognitive reserve to insults, though few studies have been able to address this directly (19). One study found that individuals without a prior diagnosis of dementia (a marker of frailty and reserve) had a worse prognosis from delirium than those with a diagnosis, supporting our finding that fitter patients may incur a worse prognosis (20). A separate finding that mortality after delirium appears to arise independently of illness severity or frailty (21). We report a similar distribution of FI scores, though our study was large enough to explore the interactions presented here.

The mechanisms by which delirium independently increases risk of death (after adjusting for illness severity) remain unclear. Delirium complicates and impairs recovery—patients with delirium are more likely to receive psychotropic drugs, more likely to fall, and less likely to mobilize effectively during and after their illness, all of which may have an adverse impact on survival (22). In addition, they may be less likely to maintain adequate hydration and malnutrition, and be less compliant with medication. Of particular concern might be hypoactive delirium, where reduced arousal might lead to aspiration pneumonia. Overall, such findings serve to emphasize the emergency nature of delirium.

To conclude, in this exploratory analysis of a large cohort of consecutive admissions to an acute adult medical unit, we found that both delirium and frailty independently increase the risk of death. In addition, the risk of death is higher in delirious patients at all levels of frailty. The most striking finding using this approach is that the risk of death from delirium is highest in the fittest patients. This highlights the crucial importance of preventing, detecting, and treating delirium in any patient, and recognizing it as a serious condition with prognostic significance.

## Supplementary Material

Supplementary data are available at *The Journals of Gerontology, Series A: Biological Sciences and Medical Sciences* online.

## Funding

The original study was funded by the Medical Research Council (UK) Special Training Fellowship in Health Services Research to E.S. D.D. is funded through a Wellcome Trust Intermediate Clinical Fellowship (WT107467).

## Conflict of interest statement

None declared.

## Supplementary Material

Supplementary Figure 1

Supplementary Legends

Supplementary Table 1
