# Supplementary figures and images for: Delirium, Frailty, and Mortality: Interactions in a Prospective Study of Hospitalized Older People
Source: J Gerontol A Biol Sci Med Sci. 2017 Nov 1;73(3):415–8. doi: 10.1093/gerona/glx214 (PMC5861945; doi:10.1093/gerona/glx214)

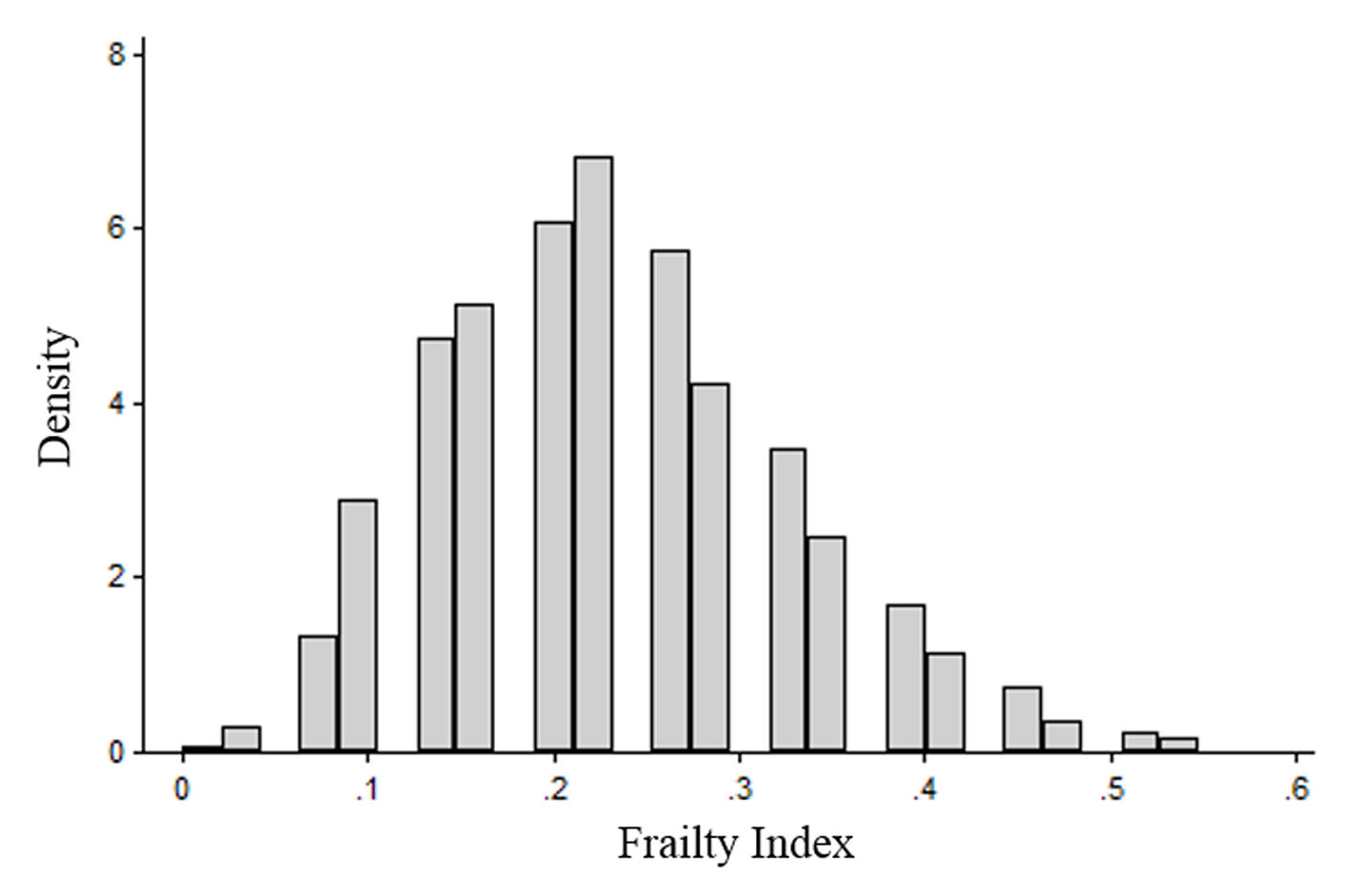

Supplement: Supplementary Figure 1 [file glx214_suppl_supplementary_figure_1.png]
