# Supplementary material for: Delirium, Frailty, and Mortality: Interactions in a Prospective Study of Hospitalized Older People
Source: J Gerontol A Biol Sci Med Sci. 2017 Nov 1;73(3):415–8. doi: 10.1093/gerona/glx214 (PMC5861945; doi:10.1093/gerona/glx214)
Supplement: Supplementary Legends [file glx214_suppl_supplementary_legends.docx]

**Supplementary Captions**

**Supplementary Table 1:** Cohort characteristics

**Supplementary Figure 1.** Distribution of a frailty index incorporating acute and chronic health factors.
