# Supplementary material for: Delirium, Frailty, and Mortality: Interactions in a Prospective Study of Hospitalized Older People
Source: J Gerontol A Biol Sci Med Sci. 2017 Nov 1;73(3):415–8. doi: 10.1093/gerona/glx214 (PMC5861945; doi:10.1093/gerona/glx214)
Supplement: Supplementary Table 1 [file glx214_suppl_supplementarytable1.doc.docx]

| Characteristic | **Frequency (%)** | |
| --- | --- | --- |
| Age, years (n=708, mean = 83.1, SD = 7.41): | | |
| 70-79 | | 38 |
| 80-89  90+ | | 43  19 |
| Sex (n=710):  Female | | 59 |
| Housing type (n =710):  House  Nursing  Residential  Sheltered  Unknown | | 72  14  7  7  1 |
| Diagnosis of dementia according to DSM-IV (n = 616): | | |
| Yes | | 42 |
| Functional assessment staging test (FAST) score (n=619): | | |
| 1: Normal ageing, no deficits  2-5: Mild cognitive impairment – moderate dementia, begins to need help with activities of daily living  6a-c: Moderately severe dementia – needs help with personal care  6d-e: Moderately severe dementia – urinary +/- faecal incontinence  7a-f: Severe dementia, loss of ability to speak, walk, sit up and smile | | 43  29  11  10  7 |
|  | | |

**Supplementary Table 1:** Cohort characteristics
